# Psoriasis en población pediátrica: un estudio descriptivo, retrospectivo y multicéntrico en Colombia

**DOI:** 10.7705/biomedica.7638

**Published:** 2025-05-30

**Authors:** Mauricio Torres, Juliana Flórez, María Salomé Páez, Ángela María Londoño, Paola Cárdenas, Mariela Tavera, Mónica Paola Novoa, Carolina Cortés, Rosángela Casanova

**Affiliations:** 1 Departamento de Dermatología, Fundación Universitaria de Ciencias de la Salud, Bogotá, D. C., Colombia Fundación Universitaria de Ciencias de la Salud Fundación Universitaria de Ciencias de la Salud Bogotá, D. C. Colombia; 2 Clínica de Psoriasis y Enfermedades Inmunomediadas - CLIPSO, Medellín, Colombia Clínica de Psoriasis y Enfermedades Inmunomediadas Clínica de Psoriasis y Enfermedades Inmunomediadas Medellín Colombia; 3 Consultorio particular, Bogotá, D.C., Colombia Consultorio particular Consultorio particular Bogotá, D.C. Colombia; 4 Departamento de Epidemiología, Fundación Universitaria de Ciencias de la Salud, Bogotá, D. C., Colombia Fundación Universitaria de Ciencias de la Salud Fundación Universitaria de Ciencias de la Salud Bogotá, D. C. Colombia

**Keywords:** psoriasis, dermatología, prevalencia, terapéutica, pediatría, niño, Colombia, Psoriasis, dermatology, prevalence, therapeutics, pediatrics, child, Colombia

## Abstract

**Introducción.:**

La psoriasis pediátrica tiene una prevalencia del 0,5 al 4,1 %. Es más frecuente en el sexo femenino y predomina su variante vulgar o en placa. Se ha relacionado con comorbilidades como la obesidad y el síndrome metabólico, e incluso, con antecedentes familiares de psoriasis.

**Objetivo.:**

Describir las características epidemiológicas y clínicas de los pacientes pediátricos con diagnóstico de psoriasis que consultaron a dermatólogos miembros de la Asociación Colombiana de Dermatología, de enero de 2017 a junio de 2022.

**Materiales y métodos.:**

Se trata de un estudio descriptivo, retrospectivo y multicéntrico, en el cual se determinó la prevalencia de la psoriasis, y se describen las características clínicas, el método diagnóstico, los antecedentes personales y familiares, y los tratamientos en casos pediátricos. Las variables se analizaron con el programa Stata 13™.

**Resultados.:**

De 26.870 pacientes pediátricos atendidos, 146 tuvieron diagnóstico de psoriasis (0,54% de prevalencia), con una relación entre mujeres y hombres de 1,5:1. La mediana de la edad de aparición fue de ocho años. La variante más común fue la vulgar (72,6 %), seguida de la *guttata* (6,85 %). El cuero cabelludo fue la localización más frecuente (55,48 %). Las comorbilidades más comunes fueron obesidad y enfermedad de Crohn (5,5 % cada una). En cuanto a antecedentes familiares, se reportaron 39 casos de psoriasis, 16 de diabetes mellitus y 7 de dislipidemia. El 78 % fueron reportados como leves, según el *Psoriasis Area Severity Index* (PASI). El 65 % de los pacientes recibió tratamiento tópico, el 18 %, sistémico y, el 2,7 %, fototerapia; en cuanto a tratamientos combinados, el 6 % recibió el tópico y sistémico, el 1,3%, el tópico más fototerapia, y el 0,68 % recibió la combinación de todos.

**Conclusiones.:**

En la población evaluada, la variante de psoriasis pediátrica más frecuente fue la vulgar en su forma leve y, en la mayoría de los casos, el tratamiento fue tópico. La asociación ocasional con otras enfermedades sistémicas, como obesidad y síndrome metabólico, implica la necesidad de ampliar los estudios.

## Abstract (en)

**Introduction.:**

Pediatric psoriasis has a prevalence ranging from 0.5 to 4.1%. It is more common in females, and the predominant type is the vulgar or plaque. It has been associated with comorbidities such as obesity and metabolic syndrome or even with a family history of psoriasis.

**Objective.:**

To describe the epidemiological and clinical characteristics of pediatric patients diagnosed with psoriasis attending pediatric dermatology consultations with dermatologists who are members of the *Asociación Colombiana de Dermatología* from January 2017 to June 2022.

**Materials and methods.:**

We conducted a descriptive, retrospective, and multicenter study. We describe the prevalence, clinical characteristics, diagnostic methods, personal and family history, and treatments for patients with pediatric psoriasis. Stata 13™ software was used for data analysis.

**Results.:**

From 26,870 patients, 146 were diagnosed with psoriasis (0.54%), with a female- to-male ratio of 1.5:1 and a median age of onset of eight years. The most common variant was vulgar psoriasis (72.6%), followed by guttate psoriasis (6.85%), and the scalp was the most frequently affected location (55.48%). The most common comorbidities were obesity and Crohn’s disease (5.5% each). Family history included 39 cases of psoriasis, 16 diabetes mellitus, and 7 dyslipidemia. Seventy-eight percent of the cases were classified as mild according to the *Psoriasis Area Severity Index* (PASI); 65% received topical treatment, and 18% received systemic treatment. Sixty-five percent received topical treatment, while systemic treatment and phototherapy was used in 19% and 2.7%, respectively. When using combined treatment (6%) received topical and systemic modalities, 1.3% used topical with phototherapy, and 0.68% received all treatments.

**Conclusion.:**

In the evaluated population, the most frequent variant of pediatric psoriasis was the vulgar type with mild severity. Most patients with this clinical presentation received topical treatment. The association with other systemic diseases implies the need for further studies.

La psoriasis es una enfermedad cutánea común, inmunomediada e inflamatoria crónica, de etiología desconocida, con origen multifactorial y predisposición genética ^1^. Existe un interés creciente en el tema debido a su asociación con comorbilidades, y a la carga social, médica, psicológica y financiera que implica para los pacientes y sus cuidadores ^2^, además del potencial que tiene para alterar la calidad de vida y el funcionamiento del individuo en general.

La prevalencia mundial de la psoriasis es de 2,0 a 3,5 % y, de estos casos, aproximadamente un tercio se inicia en la infancia, con tasas de incidencia que se han duplicado desde 1970 ^3^.

En la población pediátrica, el 4,1 % de las dermatosis corresponden a psoriasis ^2^. La psoriasis afecta aproximadamente hasta el 2 % de los niños en Europa ^4^, especialmente durante los primeros meses de vida. Sin embargo, a nivel mundial, la mediana de edad de aparición es de siete a diez años ^2^. La mayor prevalencia de psoriasis se encuentra en los países europeos, con predilección por mujeres de raza caucásica ^5^. No obstante, la información epidemiológica y clínica de la psoriasis infantil es limitada, especialmente en Latinoamérica ^4^.

Las manifestaciones clínicas de la psoriasis en la infancia son similares a las del adulto. No obstante, los pacientes pediátricos presentan con mayor frecuencia características clásicas que se destacan, como el compromiso a nivel del rostro y las zonas de flexión, las placas psoriásicas tienden a ser más pequeñas, y las escamas más finas y blandas ^6^. Las formas de presentación más frecuentes en la población pediátrica son la psoriasis en gotas (o psoriasis *guttata*) y la psoriasis palmo-plantar ^3^.

En cuanto a la asociación de la psoriasis con otras enfermedades, se ha encontrado que la población pediátrica presenta el doble de incidencia de comorbilidades asociadas, entre las cuales se encuentran la obesidad, la diabetes mellitus, la hiperlipidemia, la hipertensión y la enfermedad de Crohn ^7^^,^^8^.

El objetivo de este estudio fue describir las características epidemiológicas y clínicas de los pacientes pediátricos con diagnóstico de psoriasis que consultaron a dermatólogos de la Asociación Colombiana de Dermatología (Asocolderma), entre enero del 2017 y junio del 2022.

## Materiales y métodos

Se realizó un estudio de corte transversal, descriptivo, retrospectivo y multicéntrico. Se analizaron los datos de los pacientes pediátricos con diagnóstico de psoriasis y que asistieron a la consulta dermatológica de algunos miembros de la Asociación Colombiana de Dermatología (Asocolderma) -en el Hospital de San José, el Hospital Infantil de San José, la Clínica de Psoriasis y Enfermedades Inmunomediadas (CLIPSO), y consultorios particulares- entre enero del 2017 y junio del 2022.

Se incluyeron pacientes de 18 años o menos con diagnóstico clínico o histopatológico de psoriasis. Se identificaron los casos a partir de los códigos de la Clasificación Internacional de Enfermedades (CIE-10): L400 para psoriasis vulgar, L401 para psoriasis pustulosa generalizada, L404 para psoriasis *guttata*, L405 para artropatía psoriásica, L408 para otras psoriasis y L409 para psoriasis no especificada. La información de las variables de interés se recolectó a partir de lo reportado en la historia clínica.

Los criterios de exclusión fueron: tener 18 años cumplidos al momento del diagnóstico; tener diagnóstico de otra enfermedad relacionada con los posibles factores desencadenantes de la psoriasis u otra enfermedad que pudiera alterar la entidad de base; recibir medicamentos formulados para enfermedades diferentes que pudieran alterar la farmacodinámica o farmacocinética de los medicamentos formulados para la psoriasis, y haber tenido una gestación en el tiempo en el que recibió atención médica en las instituciones participantes.

Se obtuvieron los datos de edad, sexo, escolaridad, antecedentes personales y familiares, peso, talla, índice de masa corporal (IMC), subtipo de la enfermedad, gravedad según el PASI (*Psoriasis Area Severity Index*), compromiso ungular y articular, factores desencadenantes, presencia de comorbilidades, alteración de la calidad de vida, síntomas asociados y tratamientos categorizados como tópicos, sistémicos, fototerapéuticos o combinados.

La información se analizó con estadística descriptiva, las variables cualitativas, con frecuencias y proporciones, y las variables cuantitativas, con medidas de tendencia central y medidas de dispersión. Para calcular la prevalencia de la psoriasis, se determinó el total de pacientes pediátricos con psoriasis atendidos durante el período de recolección de datos y se dividió por el número de casos de aquellos atendidos. Los análisis se hicieron mediante el programa estadístico Stata 13™.

El presente trabajo fue sometido a valoración y aprobado por el comité de ética del Hospital San José y el Hospital Infantil de San José.

## Resultados

Entre enero del 2017 y junio del 2022, 26.870 pacientes fueron atendidos por seis dermatólogos pediatras en distintos centros médicos de Colombia. Se identificaron 146 pacientes con psoriasis confirmada por hallazgos clínicos o biopsia de piel, con una prevalencia del 0,54 %.

Respecto a las características demográficas, el 59,6 % de los casos era de sexo femenino y el 40,4 % era de sexo masculino, para una relación 1,5 a 1. La mayoría de ellos fueron atendidos en Medellín (64,4 %) y en Bogotá (35,6 %).

En cuanto a la edad de inicio, la psoriasis fue más frecuente en la infancia (51,4 %), seguida de la primera infancia y la adolescencia. La mediana de edad de presentación fue de ocho años (rango intercuartílico, RIQ = 5-10,9 años) y la mediana de edad para la primera consulta dermatológica fue de 9,6 años (RIQ = 6-12,5 años). La mayoría de los pacientes presentaron múltiples lesiones y la superficie corporal total comprometida fue menos del 10 %; únicamente el 19,1 % tuvo compromiso ungueal y el 10,3 % tuvo compromiso articular. Los métodos diagnósticos más usados fueron la histopatología y la observación clínica (cuadro 1).

**Cuadro 1:** Variantes clínicas identificadas

Variables clínicas		n (%)
Edad de inicio		
	Primera infancia (0-5 años)	42 (28,8)
	Infancia (6-11 años)	75 (51,4)
	Adolescencia (12-17 años)	29 (19,9)
Cantidad de lesiones		
	Única	11 (7,5)
	Múltiples	133 (91)
	No especificada	2 (1,4)
Superficie corporal total		
	≤ 10	134 (91,8)
	> 10	12 (8,2)
Compromiso ungueal		
	Sí	28 (19,1)
	No	24 (16,4)
	No especificado	94 (64,4)
Compromiso articular		
	Sí	15 (10,3)
	No	27 (18,5)
	No especificado	104 (71,2)
Método diagnóstico		
	Biopsia	72 (49,3)
	Clínica	55 (37,7)
	Ambos	15 (10,3)
	Ninguno	4 (2,7)

El hallazgo de infecciones fue reportado como factor desencadenante solo en tres casos, mientras que el trauma y el estrés emocional lo fueron en un caso cada uno.

**Figura 1:**
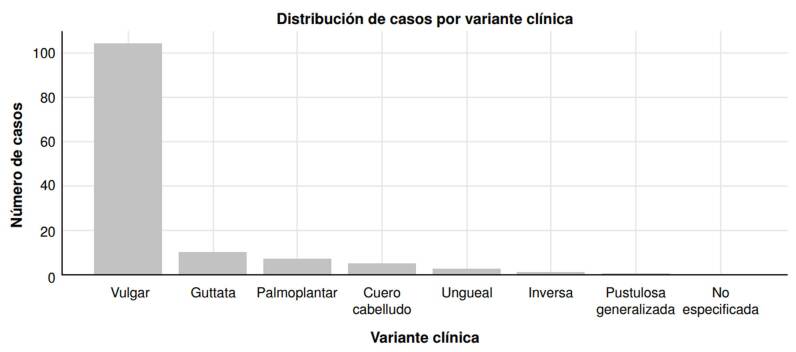
Número de casos por variante clínica de psoriasis pediátrica

La forma clínica más frecuente fue la psoriasis vulgar, que se presentó en 106 pacientes (72,6 %), seguida por la psoriasis en gotas (6,85 %), la palmoplantar (4,79 %), la del cuero cabelludo (3,42 %) y la ungueal (2,05 %) (figura 1).

**Figura 2:**
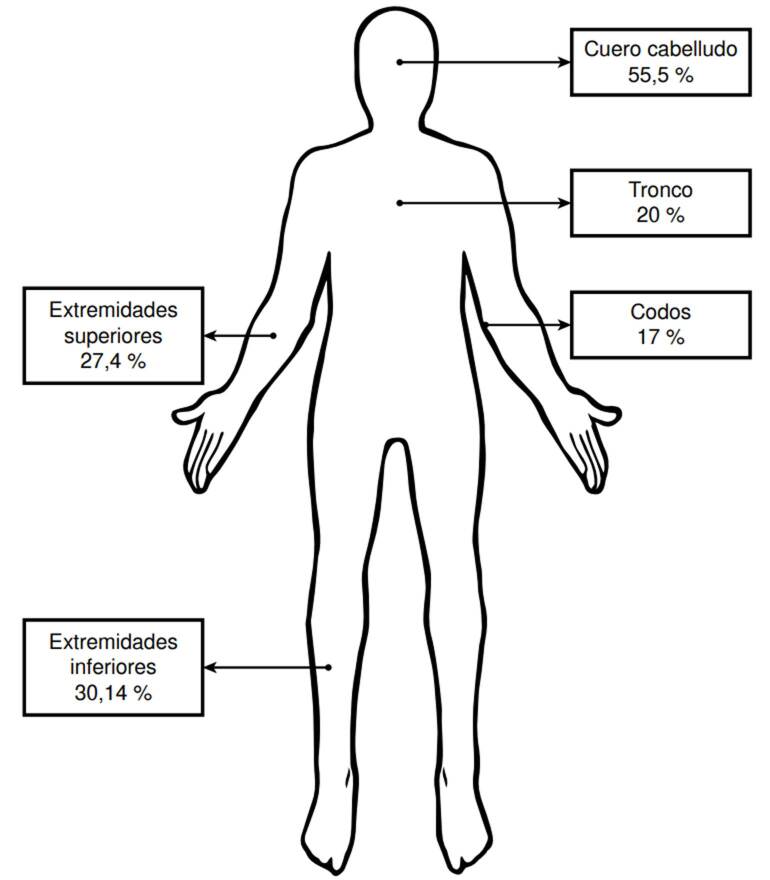
Localizaciones más frecuentes de la psoriasis pediátrica

La localización más afectada fue el cuero cabelludo (55,48 %), seguida de las extremidades inferiores (30,14 %), las extremidades superiores (27,4 %), el tronco (26 %) y los codos (17 %). En el 11 % de los casos hubo compromiso generalizado. La asociación con compromiso articular se dio en el 10 % (figura 2).

El síntoma reportado con mayor frecuencia fue el prurito (42 %), seguido del dolor articular (7 %). En más de la mitad de los casos, no se presentaron síntomas (cuadro 2).

**Cuadro 2:** Síntomas asociados

	n (%)
Sin síntomas/no especificado	77 (52,7)
Un síntoma	64 (43,8)
Prurito	58 (39,7)
Dolor articular	6 (4,1)
Dolor urente	-
Dos síntomas	5 (3,4)
Prurito y dolor articular	5 (3,4)

Las comorbilidades más frecuentes fueron la obesidad (5,5 %) y la enfermedad de Crohn (5,5 %), seguidas del síndrome metabólico y la ansiedad (2,7 % cada una). El promedio del índice de masa corporal (IMC) reportado fue de 20. No se observaron anormalidades en los exámenes paraclínicos.

En 39 casos había antecedentes familiares de psoriasis, en 16, de diabetes mellitus y, en 7, de dislipidemia. No se encontraron antecedentes familiares de artritis psoriásica, obesidad, síndrome metabólico, enfermedad de Crohn o ansiedad; se encontraron antecedentes de depresión en muy pocos casos.

La gravedad se evaluó mediante el PASI clinimetría. El PASI inicial hasta en el 78 % de los casos y el efecto en la calidad de vida en más de la mitad, fueron leves; este último, se valoró mediante el cDLQI (*Children’s Dermatology Life Quality Index*) (cuadro 3). En el 6 % se reportó ausentismo escolar. Respecto a los pacientes con compromiso ungueal, no se calculó el NAPSI (*Nail Psoriasis Severity Index*) en las historias clínicas.

**Cuadro 3:** Clinimetría

		n (%)
PASI inicial		
	Leve	114 (78,1)
	Moderada	13 (8,9)
	Grave	19 (13)
DLQI		
	Sin afectación	32 (21,9)
	Efecto leve	82 (56,2)
	Efecto moderado	18 (12,3)
	Efecto grave/muy importante	10 (6,8)
	Efecto muy grave/extremadamente importante	4 (2,7)

PASI: *Psoriasis Area Severity Index; DLQI: Dermatology Life Quality Index*

El enfoque terapéutico más utilizado fue la monoterapia con tratamiento tópico (65 %), seguido del sistémico (18 %). En este último se resalta el uso de medicamentos biológicos (6 % de los casos), como adalimumab (n = 3), etanercept (n = 3), ustekinumab (n = 2) y secukinumab (n = 1). Las modalidades tópica y sistémica se combinaron en el 6,2 % de los casos. Otra opción terapéutica fue la fototerapia, usada en el 2,7 % de los casos o, con menor frecuencia, en combinación con otros tratamientos.

## Discusión

Este estudio tuvo como finalidad describir las características epidemiológicas y clínicas de los pacientes pediátricos con diagnóstico de psoriasis que asisten a consulta dermatológica en Colombia. Los datos se obtuvieron para el periodo 2017-2022, a partir de la base de datos de dos hospitales universitarios (Hospital San José y Hospital Infantil de San José) de Bogotá, un centro de referencia de psoriasis pediátrica en Medellín (Clínica de Psoriasis y Enfermedades Inmunomediadas, CLIPSO) y de los consultorios particulares de cinco dermatólogos pediatras en Bogotá.

Según los datos obtenidos, la prevalencia de psoriasis fue del 0,54 % en la población pediátrica evaluada, lo que contrasta con lo reportado en la literatura, ya que la prevalencia a nivel mundial es del 2 % al 3,5 % ^9^ y, de estos casos, la tercera parte corresponde a pacientes pediátricos ^2^. No se encontró un reporte de prevalencia única en la población pediátrica con psoriasis a nivel mundial ni en Colombia, lo que le da más relevancia al dato que se reporta en este estudio.

La edad promedio de inicio de la psoriasis fue de ocho años, semejante a los datos reportados en la literatura mundial (mediana de aparición de 7 a 10 años) ^2^. La edad promedio de la primera consulta dermatológica fue de 9,6 años. No obstante, aunque en la literatura no se reporta este dato, esto puede ser indicativo de un retraso en el diagnóstico, debido posiblemente a diferentes factores, como el momento de consultar a dermatología o remisiones tardías por desconocimiento de la enfermedad.

En cuanto a la prevalencia por sexo, únicamente un estudio publicado en el 2013, en Estados Unidos, reportó una relación de 1,48:1 entre la aparición de psoriasis en mujeres versus hombres en pacientes pediátricos, lo que muestra una predilección de la condición por el sexo femenino ^5^. Es interesante su similitud con el presente estudio, en el cual dicha relación fue de 1,5:1.

Respecto a presentación clínica de la enfermedad, la más frecuente fue la psoriasis vulgar, seguida de la psoriasis en gotas y la palmo-plantar. Esto coincide con algunos estudios publicados de Norteamérica, en los cuales la psoriasis en placas fue la más frecuente, seguida de la psoriasis en gotas ^10^^,^^11^. Es importante mencionar que, en menores de dos años -según la guía de práctica clínica para el tratamiento de la psoriasis en Colombia-, la forma clínica más frecuente es la psoriasis en el área del pañal.

Las zonas más comprometidas fueron el cuero cabelludo, seguido de las extremidades superiores e inferiores. Esto contrasta con lo informado en una revisión bibliográfica, en la cual la mayoría de la población provenía de Europa, Asia y los Estados Unidos; en esta, el compromiso fue más frecuente en codos, rodillas y espalda baja, y fue menos frecuente en tronco, zonas distales de las extremidades y cuero cabelludo ^11^. Lo anterior pone de manifiesto la posible heterogeneidad de presentaciones clínicas entre distintas poblaciones.

El compromiso ungueal es útil para establecer la gravedad de la enfermedad y su pronóstico, especialmente, en cuanto el riesgo de desarrollar artritis psoriásica; en algunas ocasiones, es la única manifestación clínica. En este estudio, la frecuencia de compromiso ungueal fue del 2,74 %, similar a lo informado en una publicación de India (2,3 %). Sin embargo, estos datos contrastan con los de otro estudio de Kuwait (38 %), y con los de la guía de práctica clínica para el tratamiento de la psoriasis en Colombia, donde oscila entre 7 % y 40 %. Esta variación podría deberse a distintos factores, entre ellos, el origen geográfico ^12^^,^^13^. Durante la recolección de los datos, se evidenció que en algunas historias clínicas no se hizo referencia al compromiso ungueal; por ello, se debe hacer énfasis en una evaluación completa del paciente, que incluya los cambios ungueales y escalas de valoración clínica como el NAPSI.

El síntoma más frecuente fue el prurito en el 42 % de los pacientes, porcentaje que dista de lo reportado en un estudio de India en el 2004, en el cual el 87 % de los pacientes reportó dicho síntoma ^13^; en otros estudios, no especifican la frecuencia del prurito. Este hallazgo evidencia una diferencia importante con la psoriasis en pacientes adultos, la cual es prácticamente asintomática ^14^.

De los 15 casos con compromiso articular reportado, en más de la mitad no se especificó la articulación comprometida. Por esta razón, estos datos no son suficientes para obtener conclusiones respecto a la localización más usual de la enfermedad. Sin embargo, es importante mencionar que, en nueve casos, se documentó dolor articular.

Las comorbilidades más frecuentes fueron la obesidad, la enfermedad de Crohn, el síndrome metabólico y la ansiedad, aunque se encontraron en muy pocos casos. Es importante mencionar que, a nivel mundial, hay estudios ^8^^,^^10^ en que se reconoce la relación de la psoriasis con las comorbilidades mencionadas, a pesar de que su fisiopatología no se ha descrito por completo. Esto evidencia la relevancia de una anamnesis completa para determinar la necesidad de estudios complementarios y de implementar un enfoque integral; teniendo en cuenta que, en adultos, la obesidad es mucho más frecuente, prevenirla desde temprana edad podría impactar significativamente en el curso de la psoriasis.

Respecto a los antecedentes familiares, los más comunes fueron psoriasis, diabetes mellitus y dislipidemia. En una revisión de la literatura, Kang *et al*. reportaron el antecedente de psoriasis en familiares de primer grado en 49 % de los casos, mientras que, en el presente estudio, el porcentaje fue del 27 % ^11^. Sin embargo, en ambos casos, este antecedente es relevante ya que un historial familiar positivo de psoriasis puede predecir su inicio temprano, pues el 30 % de los pacientes tiene un familiar de primer grado afectado ^2^.

La clinimetría en la psoriasis desempeña un papel fundamental. Las herramientas como el PASI y el cDLQI facilitan la calificación objetiva de la gravedad de la enfermedad para lograr una estandarización universal de los hallazgos. En la literatura, la gravedad de la psoriasis tiende a definirse según el área de superficie corporal comprometida. En un estudio multicéntrico de Estados Unidos, la psoriasis se consideró leve con un área comprometida menor de 5 %, como moderada, con una de 5 a 10 % y, como grave, con una mayor del 10 % del área corporal ^5^. En el presente estudio se usó el PASI por ser el más reportado, aunque solo se registró en el 43% de las historias clínicas; en la mayoría (78,1 %), la gravedad inicial fue leve. Asimismo, el efecto sobre la calidad de vida fue leve en más de la mitad de los casos, aunque el cDLQI solo se reportó en el 61 %. Una minoría de pacientes presentó enfermedad moderada (8,9 %) o grave (13 %), por lo cual fueron candidatos a tratamiento sistémico o fototerapia.

Eichenfield *et al*., en su estudio publicado en el 2018 ^2^, encontraron que, en la evaluación de los niños con psoriasis mediante el cDLQI, el desarrollo social era el más alterado, con afectación del desempeño escolar y la salud emocional. Estas alteraciones resultaron en grandes limitaciones para realizar actividades recreativas del 15 al 30 % de los pacientes. En el presente estudio, únicamente se reportó ausentismo escolar en 6 % de los casos, aunque en gran parte de las historias clínicas no se aclaró si se había indagado al respecto; es un dato importante para estimar la afectación de la calidad de vida. Es relevante tener en cuenta que, en el cDLQI, un puntaje de 0-1 indica que no hay ningún compromiso de la calidad de vida, uno de 2-6, uno leve, uno de 7-12, uno moderado, uno de 13-18, uno grave y, uno de 1930, uno muy grave ^4^.

En cuanto al compromiso ungueal, solo en el 33 % de casos se encontró registrado el valor del NAPSI en las historias clínicas. Teniendo en cuenta la frecuencia previamente reportada del uso de otras escalas clinimétricas, es de suma importancia incentivar a los dermatólogos para que realicen y plasmen las clinimetrías en las historias clínicas, ya que, estos resultados ayudarán a sacar conclusiones sobre la evolución de la enfermedad y al inicio o modificación de los esquemas de manejo.

Como limitaciones del estudio, se identificó un sesgo de selección, debido a que la búsqueda por códigos CIE-10 en las historias clínicas no asegura que la enfermedad esté clasificada adecuadamente. A pesar de que los datos fueron recolectados en varias instituciones, estas se encuentran en las ciudades principales de Colombia, zonas donde es probable que los pacientes con barreras de acceso al sistema de salud no consulten. El carácter retrospectivo del estudio conlleva el riesgo de tener datos incompletos en la historia clínica, lo que se refleja en la dificultad para proporcionar algunos resultados o conclusiones con precisión. Además, se identificó la posibilidad de un sesgo de confusión, ya que fue necesario considerar múltiples factores asociados o desencadenantes que podrían estar relacionados entre sí o estar presentes sin tener relación alguna con el inicio de la psoriasis. Se intentó mitigar esto, buscando evidencia al respecto y estableciendo una línea de tiempo de la evolución de la enfermedad y las comorbilidades reportadas (cuando fue posible). La mayoría de los pacientes no evidenciaron alteraciones en exámenes paraclínicos.

El esquema de tratamiento más utilizado fue el tópico, lo que posiblemente está relacionado con el carácter leve de la enfermedad en la mayoría de los casos; se destacan los corticoides de mediana y gran potencia, los inhibidores de la calcineurina, la urea y los análogos de la vitamina D. A este esquema le siguieron los sistémicos, que incluyeron antihistamínicos, metotrexato, productos biológicos, y la combinación del tratamiento tópico y el sistémico. La alternativa menos utilizada fue la fototerapia. En la guía para el manejo y tratamiento de la psoriasis en pacientes pediátricos de la *National Psoriasis Fundation de la American Academy of Dermatology*, recomiendan los corticosteroides tópicos cuando la enfermedad está localizada. No hay guías formales para el uso de análogos de la vitamina D, aunque se ha visto que son seguros y bien tolerados. Aunque la antralina se ha usado con éxito, se recomienda su aplicación en el consultorio, lo que resulta dispendioso, además de su poca disponibilidad en Colombia. Las guías también indican que la fototerapia es eficaz para la psoriasis no controlada. Sin embargo, faltan estudios en pacientes pediátricos sobre los efectos secundarios potenciales de la antralina a largo plazo. En la enfermedad recalcitrante, pueden usarse tratamientos sistémicos como el metotrexato, la ciclosporina, los retinoides y los productos biológicos (antifactor de necrosis tumoral - anti-TNF e inhibidores de la interleucina 12 o 23), aunque la mayoría aún no están aprobados ^15^.

En conclusión, se encontraron múltiples coincidencias con lo reportado en otras poblaciones respecto a la edad de inicio, la mayor prevalencia de la enfermedad en el sexo femenino, la psoriasis vulgar como la forma clínica más frecuente, la poca frecuencia de compromiso ungueal, el prurito como síntoma más frecuente y los antecedentes de psoriasis en familiares de primer grado. Se encontraron diferencias significativas respecto a las localizaciones afectadas, la frecuencia de las comorbilidades y el ausentismo escolar, según lo reportado en las guías colombianas de psoriasis del 2018 y el 2022 ^16^^,^^17^.

No fue posible comparar las prevalencias únicamente en población pediátrica, por la ausencia de este dato en la literatura revisada. De hecho, aquí radica la importancia de este estudio, por ser el primero que presenta datos sobre población pediátrica colombiana con psoriasis. Además, se evidencia que estandarizar los datos que deben obtenerse en cada consulta, permite estimar la evolución de la enfermedad de una manera objetiva, ya que esta información es la base para los análisis de estudios como este.

Los resultados de esta investigación, con sus coincidencias y diferencias respecto a otras publicaciones, permiten una comprensión más amplia y un manejo integral de los pacientes pediátricos con psoriasis en Colombia. Aun así, se requieren más estudios de este tipo, inclusive prospectivos, que involucren más instituciones nacionales e internacionales, y aporten información adicional y actualizada.
